# Burden of lymphoma in China, 2006–2016: an analysis of the Global Burden of Disease Study 2016

**DOI:** 10.1186/s13045-019-0785-7

**Published:** 2019-11-19

**Authors:** Weiping Liu, Jiangmei Liu, Yuqin Song, Xinying Zeng, Xiaopei Wang, Lan Mi, Cai Cai, Lijun Wang, Jun Ma, Jun Zhu, Union for China Leukemia Investigators of the Chinese Society of Clinical Oncology, Union for China Lymphoma Investigators of the Chinese Society of Clinical Oncology

**Affiliations:** 1 Key Laboratory of Carcinogenesis and Translational Research (Ministry of Education), Department of Lymphoma, Peking University Cancer Hospital & Institute, Beijing, China; 2 National Center for Chronic and Noncommunicable Disease Control and Prevention, Chinese Center for Disease Control and Prevention, Beijing, China; 3 Beijing Institute of Survey and Mapping, Beijing Municipal Key Laboratory of Urban Spatial Information Engineering, Beijing, China; 4 Department of Hematology & Oncology, Harbin Institute of Hematology & Oncology, Harbin, China

**Keywords:** Lymphoma, Hodgkin’s disease, Lymphoma, Non-Hodgkin, Epidemiology, Incidence, Mortality, Prevalence

## Abstract

**Background:**

The accurate information about lymphoma burden at national and provincial levels remains unknown in China.

**Methods:**

Following the general analytical strategy used in GBD 2016, the age-, sex-, and province-specific incidence, mortality, and prevalence of lymphoma in China were analyzed. Trends in the incidence, mortality, prevalence, and disability-adjusted life years (DALYs) due to Hodgkin’s lymphoma (HL) and non-Hodgkin’s lymphoma (NHL) were assessed from 2006 to 2016.

**Results:**

It was estimated that there were 75,400 new cases and 40,500 deaths of lymphoma in 2016 in China, of which 6900 new cases and 2900 deaths were due to HL, while 68,500 new cases and 37,600 deaths were due to NHL. The age-standardized incidence rate (ASIR), mortality rate (ASMR), and prevalence rate (ASPR) per 100,000 were 0.46, 0.19, and 1.75 for HL, and 4.29, 2.45, and 14.9 for NHL, respectively. An upward trend with age in incidence and mortality was observed. Males had higher incidence and mortality rates than females in all age groups. Sociodemographic index had a correlation with the ASIR (*r* = 0.75), ASMR (*r* = − 0.74), ASPR (*r* = 0.84), and age-standardized DALYs (*r* = − 0.75) of HL, as well as with the ASIR (*r* = 0.80), ASPR (*r* = 0.83), and age-standardized DALYs (*r* = − 0.33) of NHL. From 2006 to 2016, the age-standardized DALYs of HL decreased significantly, while the age-standardized DALYs of NHL increased from 2006 to 2013 and remained stable from 2013 to 2016.

**Conclusions:**

The burden of lymphoma in China showed unexpected patterns varied by sex, age, and provinces, with an increased trend of NHL and a decreased trend of HL from 2006 to 2016.

**Electronic supplementary material:**

The online version of this article (10.1186/s13045-019-0785-7) contains supplementary material, which is available to authorized users.

## Background

Lymphoma, comprising Hodgkin’s lymphoma (HL) and non-Hodgkin’s lymphoma (NHL), is one of the common cancers worldwide. In 2016, the incident cases and death number were 73,000 and 28,700 due to HL globally, and 461,000 and 239,600 due to NHL, respectively [1]. Of note was that the mortality rates of HL and NHL decrease during the period of 2006–2016 [2]. According to the statistics of GLOBOCAN 2018 [3], lymphoma accounted for 3.2% of the 18.1 million new cancer cases (0.4% due to HL and 2.8% due to NHL) and 2.9% of the 9.6 million cancer deaths worldwide in 2018 (0.3% due to HL and 2.6% due to NHL). Compared with the statistics of GLOBOCAN 2012 [4], both new cases and deaths associated with lymphoma increased in 2018.

The disease burden of lymphoid neoplasms has been rising in China over the last decade [2]. Based on the data from the National Central Cancer Registry of China [5], it was estimated that lymphoma and myeloma accounted for 2.1% (88,200 new cases) of all new cancer cases and 1.9% (52,100 deaths) of all cancer deaths in 2015. A recent study [6] showed that the mortality rates of lymphoma and myeloma increased annually by 4.5% during the period 2004–2016. However, accurate epidemiologic information of lymphoma based on national population cannot be available in China. Ultimately, an understanding of trends will help to direct future studies on strategy for disease control and prevention. Therefore, this analysis aimed to determine the incidence, mortality, and prevalence of lymphoma in 2016 and analyze temporal trends from 2006 to 2016 in China.

## Methods

### Data sources

The Global Burden of Disease 2016 (GBD 2016) study, which covered 195 countries and territories between 1990 and 2016, provided a comprehensive assessment of health loss for 333 diseases and injuries [7]. Details of the methodology used in the GBD 2016 study have been explained in previous studies [2, 8, 9]. The present study focused on the burden of lymphoma nationally in China. The main mortality data came from the disease surveillance point system and the cause of death reporting system from the Chinese Center for Disease Control and Prevention. Incidence data were sought from individual cancer registries or aggregated databases of cancer registry data like “Cancer Incidence in Five Continents (CI5).” All metadata for each source retrieved through the GBD 2016 database were available in the online GBD citation tool (http://ghdx.healthdata.org/gbd-2016). In addition, a total of 33 province-level administrative units were analyzed in this study, including 31 mainland provinces, municipalities, and autonomous regions, and the Hong Kong and Macao special administrative regions. International Classification of Diseases-10 (ICD-10) codes were used to represent HL (C81–C81.99) and NHL (C82–C86.6, C96–C97.9).

### Estimates of disease burden

The general methodological approaches used for GBD 2016 have been described elsewhere [1, 2, 7, 8]. In summary, the methodological framework started with estimating mortality. Various data from different sources were procured to generate cause-specific mortality estimates by the use of Cause of Death Ensemble Model (CODEm). During the process, the incidence and mortality data from different sources generated the crude mortality-to-incidence ratios (MIR) using a linear-step mixed-effects model with the sociodemographic index (SDI), a composite indicator of income, education, and fertility, as the predictive covariate, and then arrived at the final MIR using the general spatiotemporal Gaussian process regression (ST-GPR). Incidence estimates were generated by dividing final mortality estimates by the MIR, and 10-year prevalence estimates were modeled using the MIR and the survival estimates. Years of life lost (YLLs) were estimated by multiplying the estimated number of deaths by patient age with a standard life expectancy at the corresponding age. Years lived with disability (YLDs) were estimated by multiplying the prevalence with a distinct disability weight in a Bayesian regression model (Dismod-MR 2.1). Disability-adjusted life years (DALYs) were calculated by summing up the YLLs and YLDs, which were used to measure the loss of health due to both fatal and non-fatal disease burden.

### Statistics

The burden of lymphoma was represented by age-standardized incidence rates (ASIRs), age-standardized mortality rates (ASMRs), age-standardized prevalence rates (ASPRs) and age-standardized mortality rate DALYs. Age-standardized rates were calculated by the GBD world population. To explore the effect of the development status on the lymphoma burden, we analyzed the association between SDI and the ASIR, ASMR, ASPR, and age-standardized DALYs. The 95% uncertainty interval (UI) for each quantity was used in the analyses.

Temporal trends in lymphoma burden from 2006 to 2016 were examined by IBM SPSS Statistics for Windows (version 21.0; IBM Corp) and fitting joinpoint models (version 4.6.0.0; National Cancer Institute). The trends were expressed as annual percentage changes (APCs), and *Z* tests were used to assess whether the APCs were significantly different from zero. In describing trends, the terms “increase” and “decrease” were used when the slope of the trend was statistically significant; otherwise, the term “stable” was used. Statistical significance was assessed at the 0.05 level, and all hypothesis tests were two-sided.

## Results

### Lymphoma burden in China, 2016

It was estimated that there were 6900 new cases of HL and 68,500 of NHL in China, accounting for 9.5% of HL and 14.9% of NHL in the world, respectively. The ASIR of HL and NHL per 100,000 was 0.46 and 4.29, respectively (Table 1). It was estimated that 2900 deaths of HL and 37,600 deaths of NHL occurred in China, accounting for 10.1% deaths of HL and 15.7% of NHL in the world, respectively. The ASMR of HL and NHL per 100,000 was 0.19 and 2.45, respectively. The estimated number of HL and NHL cases was 26,000 and 237,000 in China, accounting for 8.9% of HL and 14.2% of NHL in the world, respectively. The ASPR of HL and NHL per 100,000 was 1.75 and 14.9, respectively. The number of DALYs and the age-standardized DALYs per 100,000 population were 90,580.66 and 5.95 for HL, and 1,097,314.48 and 70.67 for NHL, respectively.

**Table 1:** Age-standardized incidence, mortality, prevalence, YLL, YLD, and DALY rates in 2016

Variable	Hodgkin’s lymphoma		Non-Hodgkin’s lymphoma	
	Numbers (thousand)	Age-standardized rates (per 100,000)	Numbers (thousand)	Age-standardized rates (per 100,000)
Incidence	6.95 (5.50–8.66)	0.46 (0.36–0.57)	68.50 (57.30–72.35)	4.29 (3.61–4.52)
Mortality	2.92 (2.40–3.79)	0.19 (0.16–0.25)	37.64 (31.36–40.23)	2.45 (2.06–2.61)
Prevalence	26.36 (20.37–32.38)	1.75 (1.35–2.13)	237.61 (199.02–251.96)	14.94 (12.64–15.82)
YLLs	87.75 (73.50–115.04)	5.77 (4.79–7.61)	1,074.94 (895.46–1150.27)	69.27 (58.65–73.96)
YLDs	2.83 (1.91–3.88)	1.86 (1.27–2.55)	22.37 (15.39–30.39)	1.40 (0.96–1.89)
DALYs	90.58 (75.80–118.58)	5.95 (4.94–7.83)	1,097.31 (915.34–1172.83)	70.67 (59.87–75.38)

*DALYs* disability-adjusted life years, *YLDs* years lived with disability, *YLLs* years of life lost

Data in parentheses are 95% uncertainty intervals

Compared with that in the world, the ASIR, ASMR, ASPR, and age-standardized DALYs of HL in China were significantly lower with 3- to 5-fold differences. Similarly, the ASIR, ASMR, ASPR, and age-standardized DALYs of NHL in China were lower with 1.5- to 2-fold differences (Table 2).

**Table 2:** Comparison of age-standardized incidence, mortality, prevalence, YLL, YLD, and DALY rates of lymphoma between in China and in the world, 2016

Variable (per 100,000)	Hodgkin’s lymphoma		Non-Hodgkin’s lymphoma	
	China	World	China	World
ASIR	0.46 (0.36–0.57)	1.33 (1.16–1.56)	4.29 (3.61–4.52)	6.34 (6.24–6.44)
ASMR	0.19 (0.16–0.25)	0.43 (0.36–0.50)	2.45 (2.06–2.61)	3.24 (3.18–3.30)
ASPR	1.75 (1.35–2.13)	8.63 (7.48–10.29)	14.94 (12.64–15.82)	30.75 (30.28–31.23)
Age-standardized YLLs	5.77 (4.79–7.61)	17.66 (14.77–20.84)	69.27 (58.65–73.96)	89.56 (87.26–91.73)
Age-standardized YLDs	0.18 (0.13–0.26)	0.66 (0.46–0.90)	1.40 (0.96–1.89)	2.51 (1.83–3.29)
Age-standardized DALYs	5.95 (4.94–7.83)	18.32 (15.36–21.62)	70.67 (59.87–75.38)	92.07 (89.58–94.40)

*ASIR* age-standardized incidence rate, *ASMR* age-standardized mortality rate, *ASPR* age-standardized prevalence rate, *DALYs* disability-adjusted life years, *YLDs* years lived with disability, *YLLs* years of life lost

Data in parentheses are 95% uncertainty intervals

### Lymphoma burden stratified by age and sex in 2016

**Fig. 1:**
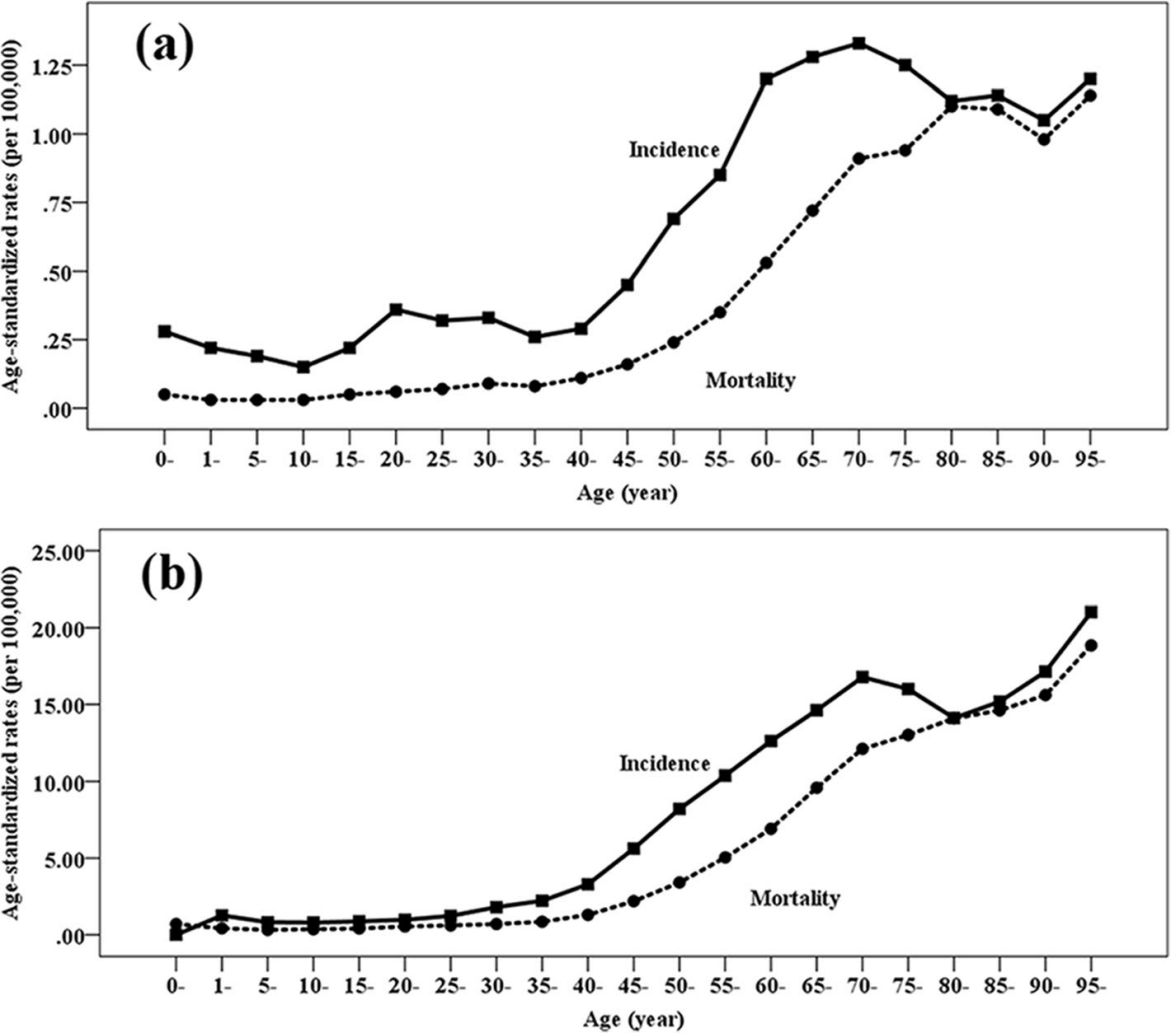
Age-standardized incidence and mortality rates of Hodgkin’s lymphoma (**a**) and non-Hodgkin’s lymphoma (**b**) by age and sex in China, 2016

Higher incidence rates of HL and NHL were seen in older individuals (Fig. 1, Additional file 1: Table S1). The ASIR of HL was less than 1 per 100,000 population in age groups less than 60 years, increased gradually with age, and reached a peak at the age group of 70–74 years (1.33 per 100,000 population). The same patterns of ASIR were seen in NHL. The ASIR of NHL was less than 5 per 100,000 population at age groups less than 45 years, increased to 10 per 100,000 population by age 65–69 years, and reached a peak at the age group of over 95 years (28.13 per 100,000 population). Males had higher ASIR than females in all age groups. After the age of 40 years, the ASIR of both HL and NHL was substantially higher in males than in females. Especially, males at the age group of 60–64 years had four times greater risk of HL than females, and males at the age group of 55–59 years had approximately two times greater risk of NHL than females.

Similar to the trends in the ASIR, an upward trend with age in the ASMR for both HL and NHL was observed (Fig. 1, Additional file 1: Table S2). The ASMR of HL increased to more than 1 per 100,000 population at the age group of 80–84 years and reached a peak at the age group of over 95 years (1.14 per 100,000 population). The ASMR of NHL increased to more than 1 per 100,000 population at the age group of 40–44 years, increased to more than 10 per 100,000 population at the age group of 70–74 years, and reached a peak at the age group of over 95 years (18.85 per 100,000 population). The ASMR in males was higher than that in females in all age groups. After the age of 40 years, the ASMR of both HL and NHL was significantly higher in males than in females. Especially, males at age groups of 45–49, 60–64, and 70–74 years had 2.5 times greater death risk of HL than females, and males at age groups of 50–54 and 55–59 years had approximately 2.5 times greater death risk of NHL than females.

### Lymphoma burden stratified by provinces in 2016

**Fig. 2:**
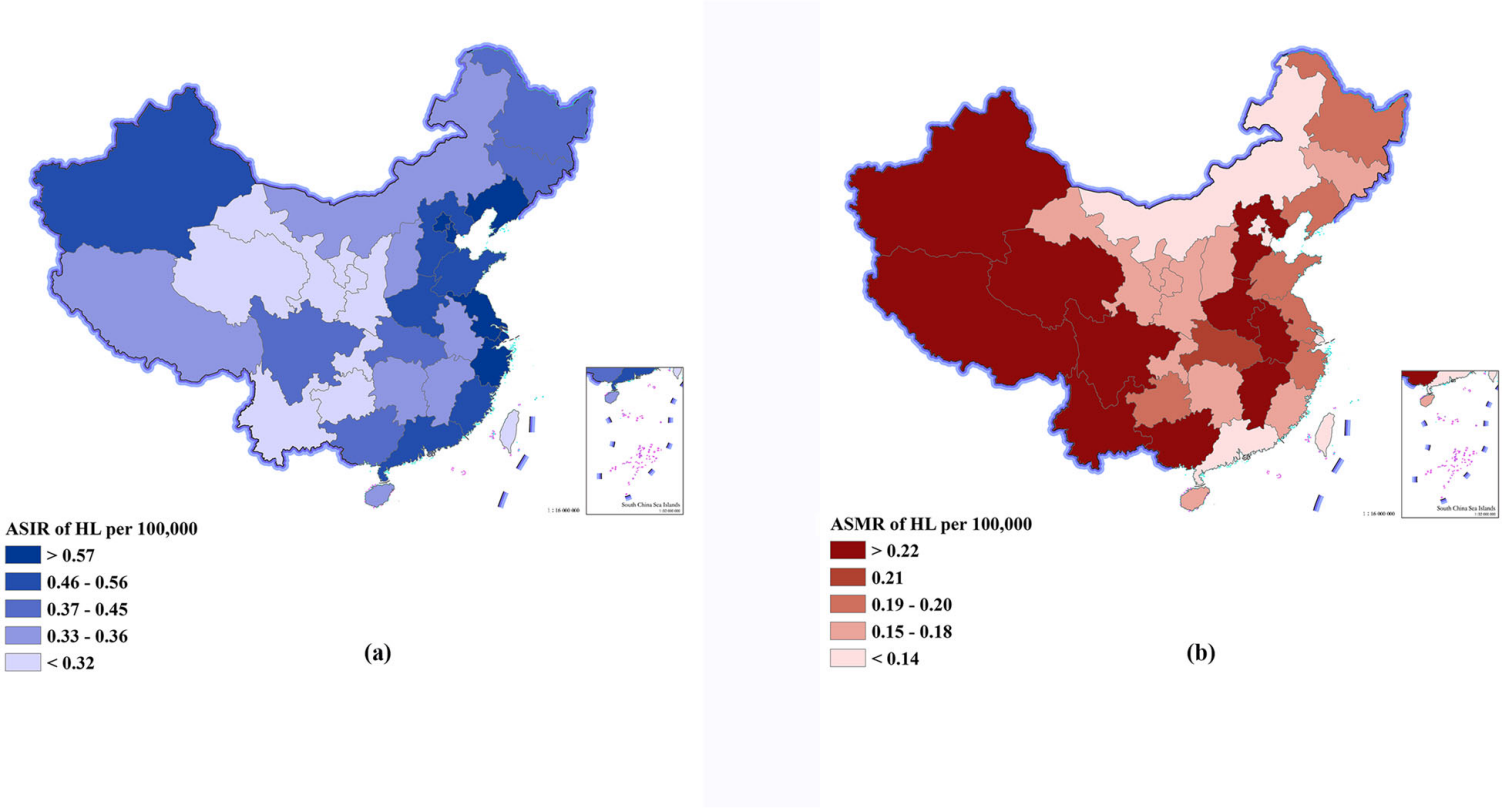
Age-standardized incidence rates (ASIRs) and age-standardized mortality rates (ASMRs) of Hodgkin’s lymphoma (HL) by province of China, 2016. **a** ASIR of HL. **b** ASMR of HL

**Fig. 3:**
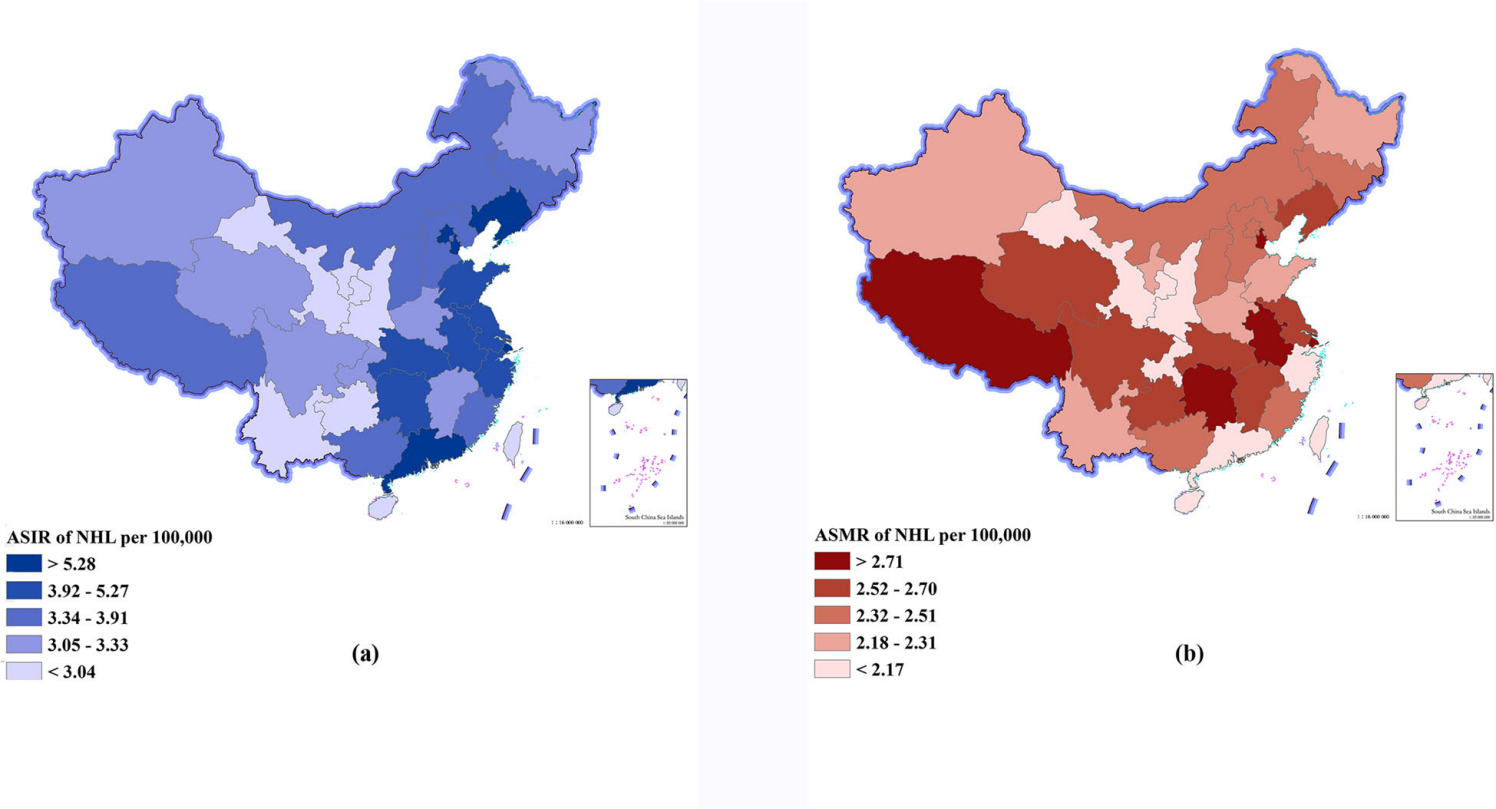
Age-standardized incidence rates (ASIRs) and age-standardized mortality rates (ASMRs) of non-Hodgkin’s lymphoma (NHL) by province of China, 2016. **a** ASIR of NHL. **b** ASMR of NHL

Figure 2, Fig. 3, and Additional file 1: Table S3 show the ASIR, ASMR, and ASPR of the 33 provinces in 2016. Totally, higher incidence and prevalence rates for both HL and NHL were seen in those provinces with high SDI, and higher DALYs for both HL and NHL were observed in those provinces with low SDI (Additional file 1: Figure S1). For HL, SDI had a strong correlation with the ASIR (*r* = 0.75, *P* < 0.01), ASMR (*r* = − 0.74, *P* < 0.001), ASPR (*r* = 0.84, *P* < 0.01), and age-standardized DALYs (*r* = − 0.75, *P* < 0.01). For NHL, SDI had a strong correlation with the ASIR (*r* = 0.80, *P* < 0.001) and ASPR (*r* = 0.83, *P* < 0.01), a weak correlation with the age-standardized DALYs (*r* = − 0.33, *P* = 0.06), and no correlation with the ASMR (*r* = − 0.07, *P* = 0.70).

For HL, the highest ASIR was observed in those provinces with high SDI such as Beijing (0.77 per 100,000), Tianjin (0.69 per 100,000), and Shanghai (0.66 per 100,000), which showed 2- to 3-fold differences compared with the lowest ASIR in those provinces with low SDI such as Ningxia (0.27 per 100,000), Gansu (0.24 per 100,000), and Guizhou (0.24 per 100,000) (Fig. 2a, Additional file 1: Figure S1A). The highest ASMR was observed in those provinces with low SDI such as Tibet (0.33 per 100,000), Hebei (0.24 per 100,000), and Xinjiang (0.24 per 100,000), which showed 2- to 5-fold differences compared with the lowest ASMR in those provinces with high SDI such as Shanghai (0.10 per 100,000), Hong Kong special administrative region (0.09 per 100,000), and Macao special administrative region (0.06 per 100,000) (Fig. 2b, Additional file 1: Figure S1B). The highest ASPR was observed in those provinces with high SDI such as Beijing (3.50 per 100,000), Tianjin (3.02 per 100,000), and Shanghai (2.95 per 100,000), which showed 3- to 5-fold differences compared with the lowest ASPR in those provinces with low SDI such as Ningxia (0.90 per 100,000), Gansu (0.73 per 100,000), and Guizhou (0.70 per 100,000) (Additional file 1: Figure S1C). The highest age-standardized DALYs were observed in those provinces with low SDI such as Tibet (11.02 per 100,000), Xinjiang (7.82 per 100,000), and Hebei (7.51 per 100,000), which showed 2- to 5-fold differences compared with the lowest age-standardized DALYs in those provinces with high SDI such as Shanghai (3.25 per 100,000), Hong Kong special administrative region (2.66 per 100,000), and Macao special administrative region (1.83 per 100,000) (Additional file 1: Figure S1D).

For NHL, the highest ASIR was observed in those provinces with high SDI such as Shanghai (8.36 per 100,000), Hong Kong special administrative region (7.78 per 100,000), and Tianjin (7.57 per 100,000), which showed 2- to 3-fold differences compared with the lowest ASIR in those provinces with low SDI such as Ningxia (2.81 per 100,000), Yunnan (2.71 per 100,000), and Gansu (2.51 per 100,000) (Fig. 3a, Additional file 1: Figure S1E). The highest ASMR was observed in those provinces with low SDI such as Tibet (3.49 per 100,000) and those with high SDI such as Hong Kong special administrative region (3.43 per 100,000), which showed 1.5-fold differences compared with the lowest ASMR in those provinces with high SDI such as Guangdong (2.06 per 100,000) and those with low SDI such as Hainan (2.06 per 100,000) (Fig. 3b, Additional file 1: Figure S1F). The highest ASPR was observed in those provinces with high SDI such as Shanghai (33.47 per 100,000), Beijing (30.31 per 100,000), and Tianjin (29.92 per 100,000), which showed 4- to 5-fold differences compared with the lowest ASPR in those provinces with low SDI such as Yunnan (7.87 per 100,000), Guizhou (7.86 per 100,000), and Gansu (7.21 per 100,000) (Additional file 1: Figure S1G). The highest age-standardized DALYs were observed in those provinces with low SDI such as Tibet (113.43 per 100,000), Hunan (87.09 per 100,000), and Anhui (86.35 per 100,000), which showed 1.5- to 2-fold differences compared with the lowest age-standardized DALYs in those provinces with high SDI such as Guangdong (59.80 per 100,000), Zhejiang (59.37 per 100,000), and Macao special administrative region (57.44 per 100,000) (Additional file 1: Figure S1H).

### Trends in lymphoma burden from 2006 to 2016

**Fig. 4:**
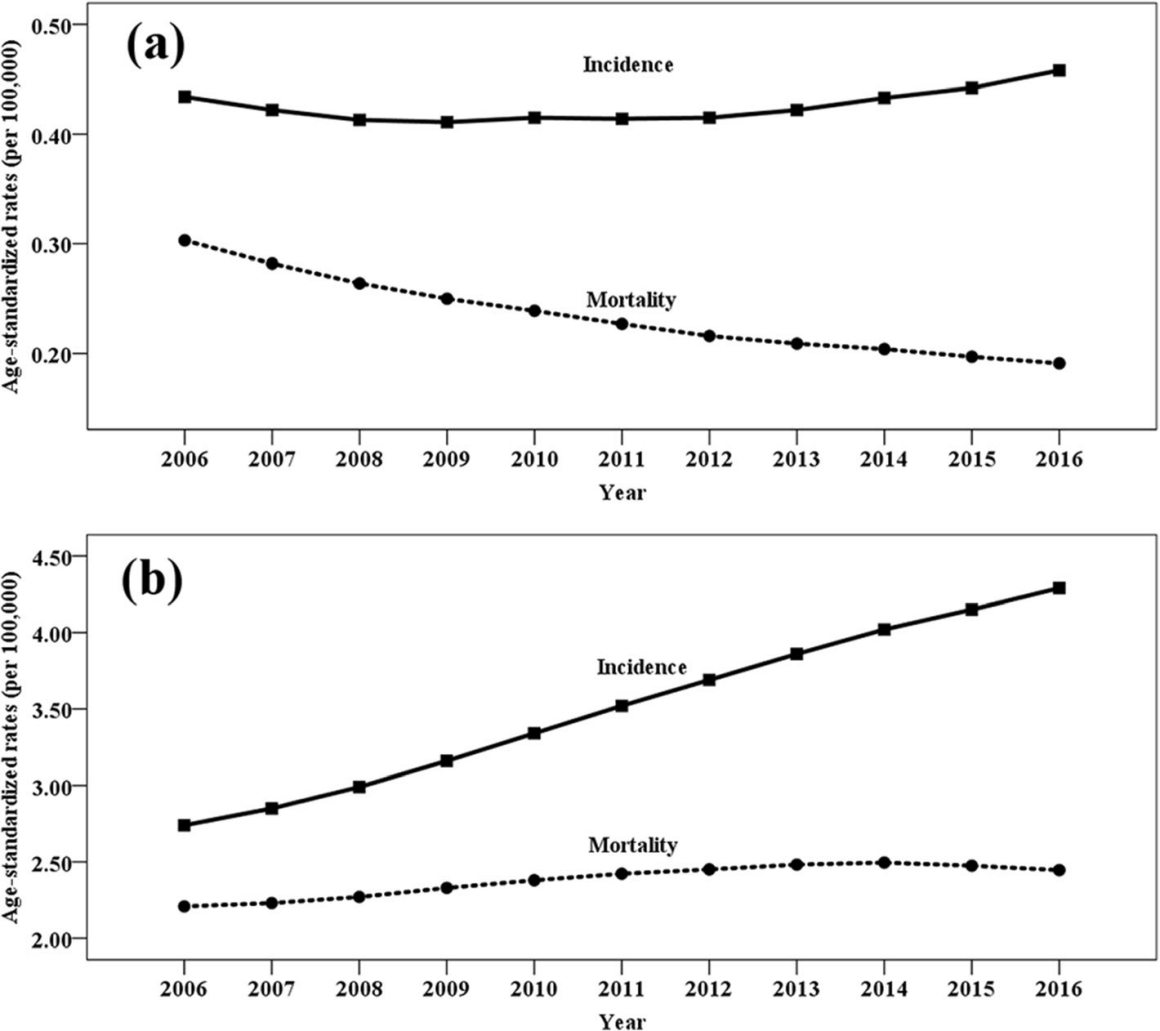
Trends in age-standardized incidence and mortality rates of Hodgkin’s lymphoma (**a**) and non-Hodgkin’s lymphoma (**b**) from 2006 to 2016

As shown in Table 3 and Fig. 4, the ASIR of HL decreased from 2006 to 2011, and then increased from 2011 to 2016, while the ASMR and DALYs decreased significantly. The ASIR of NHL increased significantly from 2006 to 2016, while the ASMR and DALYs of NHL increased from 2006 to 2013 and then remained stable from 2013 to 2016.

**Table 3:** Trends in lymphoma burden from 2006 to 2016

Variable	Hodgkin’s lymphoma				Non-Hodgkin’s lymphoma			
	Trend 1^#^		Trend 2^#^		Trend 1^#^		Trend 2^#^	
	Year	APC	Year	APC	Year	APC	Year	APC
Incidence	2006–2011	− 0.96^*^	2011–2016	2.17^*^	2006–2013	5.19^*^	2013–2016	3.47^*^
Mortality	2006–2011	− 5.63^*^	2011–2016	− 3.20^*^	2006–2013	1.84^*^	2013–2016	− 0.54
Prevalence	2006–2011	1.00^*^	2011–2016	3.92^*^	2006–2013	6.74^*^	2013–2016	4.88^*^
YLLs	2006–2012	− 5.75^*^	2012–2016	− 3.14^*^	2006–2013	1.60^*^	2013–2016	− 0.78
YLDs	2006–2011	− 1.01^*^	2011–2016	2.13^*^	2006–2013	5.34^*^	2013–2016	3.73^*^
DALYs	2006–2012	− 5.66^*^	2012–2016	− 2.98^*^	2006–2013	1.66^*^	2013–2016	− 0.69

*APC* annual percentage change, *DALYs* disability-adjusted life years, *YLDs* years lived with disability, *YLLs* years of life lost

^*^APC is significantly different from zero

^#^Each change in magnitude and/or direction of trend is listed separately with the years for which that trend was constant. Therefore, if only one trend is listed for 2006 through 2016, that trend was constant during the entire time period

## Discussion

The present study was the most comprehensive evaluation of the large and ever-growing burden of lymphoma in China. The standardized methods for estimates of lymphoma metrics used in the GBD study made it possible to compare a global level with that in China. We determined the disease burden of lymphoma in China. Over 260,000 Chinese people suffered from lymphoma in 2016, meaning that there were about 20 lymphoma patients per 100,000 population in China.

The patterns of burden of HL and NHL varied by age and sex in our study. Similar to most cancers in China, there was a strong trend for higher incidence and mortality of both HL and NHL in older individuals. Notably, lymphoma has been proven to be the third most common cancer type in males and females aged 0–14 years [10]. Male predominance of incidence and mortality was seen in all age groups for both HL and NHL, which could be explained partly by some risk factors such as smoking and infections [11]. In addition, our study highlights important variations in the geographical differences across provinces. Higher incidence rates were seen in those provinces with high SDI, and higher mortality rates were observed in those provinces with low SDI, which was mainly due to an imbalance in socioeconomic development. Based on these disparities in disease burden, different strategies for disease prevention and control should be employed when health policy is made in the future.

Although the ASIR of HL increased in China while decreased globally (6.98% vs. − 6.80%) between 2006 and 2016, the ASMR declined more rapidly in China than globally (− 35.67% vs. − 22.40%), indicating that more DALYs were saved in China. This rapid declining trend of ASMR could be associated with continuously improving diagnosis and treatment techniques. Numerous treatment options have emerged for HL, especially in the last five decades. Until now, the majority of lymphoma patients can be cured with a conventional combination of chemotherapy and radiotherapy. Salvage therapy represented by stem cell transplantation and novel agents such as brentuximab vedotin [12] and immune checkpoint inhibitors [13] may give the second chance of cure for those with relapsed or refractory disease.

The burden of NHL in China rose more significantly than globally. The change of age-standardized DALYs per 100,000 population for NHL from 2006 to 2016 was 9.18% in China and 1.4% worldwide. The prognosis of B cell NHL, the most common type of NHL, was improved by immunochemotherapy based on rituximab and anthracyclines [14]. A study from Surveillance, Epidemiology, and End Results database demonstrated that there were 279,704 cumulative life years saved and an incremental economic gain of $16.52 billion after the introduction of rituximab into clinical practice [15]. However, the protection offered by health insurance was often incomplete [16]. Many patients with NHL, especially in less-developed provinces in China, could not afford expensive anticancer agents such as rituximab. To solve this problem, health insurance reform with an aim of wider coverage was performed in China since 2009 [17], of which both equality and efficacy were taken into account [18]. Our findings highlight the need to address universal health coverage in China, and further study to compare the cost-effectiveness between pre- and post-health insurance reform should be executed.

There are marked differences in the epidemiological characteristics of lymphoma between western and eastern countries [19]. In the USA, HL incidence had a peak at the age group of 21–30 years, regardless of race [20]. Differently, our study showed HL incidence had an upward trend with age and had a peak at the age group of 70–74 years in China. Different temporal trend patterns were also observed. In the USA, incidence rates of NHL almost doubled during 1974–2009, increasing rapidly through the early 1990s, followed by more gradual increases and stable rates since the early 2000s [21]. However, the incidence rates of both HL and NHL have been increasing substantially in Asian countries such as Japan [22] and Korea [23]. Consistent with Asian reports, our study showed that the incidence of HL and NHL in China increased without any plateau from 2006 to 2016. The dramatic increase in lymphoma burden may be interpreted partly by improvements in diagnostic procedures [24] and changes of lifestyle [25, 26], but much of this trend was largely unexplained and warrants investigation.

Although the etiology of lymphoma is not yet completely understood, there are a few well-established risk factors of lymphoma such as aging, family history, and various infections. Aging is found to be the leading risk factor of lymphoma with higher incidence and mortality rates in older individuals. An upward trend with age in the ASIR and ASMR for both HL and NHL was observed (Fig. 1). The Epstein-Barr virus (EBV) and hepatitis B virus (HBV) were endemic with the seroprevalence of 90% [27] and 7.2% [28] in China, which may play a crucial role in the disease burden of lymphoma [11]. A pooled study [29] based on three prospective cohorts from China and Singapore confirmed that an increased risk of NHL was related to EBV infection (odds ratio (OR) = 2.17) and HBV infection (OR = 2.16). Similarly, the burden of HL could be attributable to EBV infection with the population-attributable fractions of 56.0% in China [30].

The interpretation of our study has several limitations. First, all the general limitations described by the GBD collaboration group [2, 7–9] apply to the present study, because the data for this study were from GBD 2016. Second, the incidence and mortality rates of HL estimated using the standard GBD methodology were very low, especially in province levels, leading to the inaccuracy of estimated results. Third, population growth and socioeconomic structures should also be considered in a cautious interpretation of the change trends of lymphoma burden in China.

## Conclusions

This is a study presenting the spatiotemporal variation in lymphoma burden in China nationally. Higher incidence and mortality rates were observed in males and older individuals. Higher incidence rates were seen in developed provinces, while higher mortality rates were seen in less-developed provinces. The difference in the disease burden between HL and NHL was also notable. The study findings provided information on the burden of lymphoma and allowed monitoring of temporal trends in the Chinese population, which will be useful for policy-making with respect to disease prevention and the development of management strategies.

## Additional file

Additional file 1: **Table S1**. Age-standardized incidence rates of lymphoma by age and sex in 2016 (per 100,000). **Table S2**. Age-standardized mortality rates of lymphoma by age and sex in 2016 (per 100,000). **Table S3**. Age-standardized incidence, mortality and prevalence rates of lymphoma by province of China in 2016 (per 100,000). **Figure S1**. Relation between lymphoma burden and socio-demographic index (SDI) at provincial level (A) age-standardized incidence rate (ASIR) of Hodgkin lymphoma (HL) vs. SDI, (B) age-standardized mortality rate (ASMR) of HL vs. SDI, (C) age-standardized prevalence rate (ASPR) of HL vs. SDI, (D) age-standardized disability-adjusted life years (DALYs) of HL vs. SDI, (E) ASIR of non-Hodgkin lymphoma (NHL) vs. SDI, (F) ASMR of NHL vs. SDI, (G) ASPR of NHL vs. SDI, (H) age-standardized DALYs of NHL vs. SDI. (PDF 687 kb)

## Data Availability

The data that support the findings of this study are available from the Chinese Center for Disease Control and Prevention, but restrictions apply to the availability of these data, which were used under license for the current study, and, thus, are not publicly available. However, data are available from the authors upon reasonable request and with permission of the Chinese Center for Disease Control and Prevention.

## Abbreviations

APCs: Annual percentage changes
ASIR: Age-standardized incidence rate
ASMR: Age-standardized mortality rate
ASPR: Age-standardized prevalence rate
DALYs: Disability-adjusted life years
GBD: Global Burden of Disease
HL: Hodgkin’s lymphoma
ICD-10: International Classification of Diseases-10
NHL: Non-Hodgkin’s lymphoma
UI: Uncertainty interval
YLDs: Years lived with disability
YLLs: Years of life lost

## References

[1] Fitzmaurice C, Akinyemiju TF, Al Lami FH, Alam T, Alizadeh-Navaei R, Global Burden of Disease Cancer Collaboration (2018). global, regional, and national cancer incidence, mortality, years of life lost, years lived with disability, and disability-adjusted life-years for 29 cancer groups, 1990 to 2016: a systematic analysis for the Global Burden of Disease Study. JAMA Oncol..

[2] GBD 2016 Causes of Death Collaborators (2017). Global, regional, and national age-sex specific mortality for 264 causes of death, 1980-2016: a systematic analysis for the Global Burden of Disease Study 2016. Lancet.

[3] Bray F, Ferlay J, Soerjomataram I, Siegel RL, Torre LA, Jemal A (2018). Global cancer statistics 2018: GLOBOCAN estimates of incidence and mortality worldwide for 36 cancers in 185 countries. CA Cancer J Clin..

[4] Torre LA, Bray F, Siegel RL, Ferlay J, Lortet-Tieulent J, Jemal A (2015). Global cancer statistics, 2012. CA Cancer J Clin..

[5] Chen W, Zheng R, Baade PD, Zhang S, Zeng H, Bray F (2016). Cancer statistics in China, 2015. CA Cancer J Clin..

[6] Liu W, Liu J, Song Y, Wang X, Zhou M, Wang L (2019). Mortality of lymphoma and myeloma in China, 2004-2017: an observational study. J Hematol Oncol..

[7] GBD 2016 DALYs and HALE Collaborators (2017). Global, regional, and national disability-adjusted life-years (DALYs) for 333 diseases and injuries and healthy life expectancy (HALE) for 195 countries and territories, 1990-2016: a systematic analysis for the Global Burden of Disease Study 2016. Lancet.

[8] GBD 2016 Disease and Injury Incidence and Prevalence Collaborators (2017). Global, regional, and national incidence, prevalence, and years lived with disability for 328 diseases and injuries for 195 countries, 1990-2016: a systematic analysis for the Global Burden of Disease Study 2016. Lancet.

[9] GBD 2016 Risk Factors Collaborators (2017). Global, regional, and national comparative risk assessment of 84 behavioural, environmental and occupational, and metabolic risks or clusters of risks, 1990-2016: a systematic analysis for the Global Burden of Disease Study 2016. Lancet.

[10] Chen W, Sun K, Zheng R, Zeng H, Zhang S, Xia C (2018). Cancer incidence and mortality in China, 2014. Chin J Cancer Res..

[11] Islami F, Chen W, Yu XQ, Lortet-Tieulent J, Zheng R, Flanders WD (2017). Cancer deaths and cases attributable to lifestyle factors and infections in China, 2013. Ann Oncol..

[12] Younes A, Bartlett NL, Leonard JP, Kennedy DA, Lynch CM, Sievers EL (2010). Brentuximab vedotin (SGN-35) for relapsed CD30-positive lymphomas. N Engl J Med..

[13] Ansell SM, Lesokhin AM, Borrello I, Halwani A, Scott EC, Gutierrez M (2015). PD-1 blockade with nivolumab in relapsed or refractory Hodgkin’s lymphoma. N Engl J Med..

[14] Howlader N, Mariotto AB, Besson C, Suneja G, Robien K, Younes N (2017). Cancer-specific mortality, cure fraction, and noncancer causes of death among diffuse large B-cell lymphoma patients in the immunochemotherapy era. Cancer..

[15] Danese MD, Reyes CM, Gleeson ML, Halperin M, Skettino SL, Mikhael J (2016). Estimating the population benefits and costs of rituximab therapy in the United States from 1998 to 2013 using real-world data. Med Care..

[16] Jan S, Laba TL, Essue BM, Gheorghe A, Muhunthan J, Engelgau M (2018). Action to address the household economic burden of non-communicable diseases. Lancet..

[17] Meng Q, Xu L, Zhang Y, Qian J, Cai M, Xin Y (2012). Trends in access to health services and financial protection in China between 2003 and 2011: a cross-sectional study. Lancet..

[18] Meng Q, Fang H, Liu X, Yuan B, Xu J (2015). Consolidating the social health insurance schemes in China: towards an equitable and efficient health system. Lancet..

[19] Armitage JO, Gascoyne RD, Lunning MA, Cavalli F (2017). Non-Hodgkin lymphoma. Lancet..

[20] Shenoy P, Maggioncalda A, Malik N, Flowers CR (2011). Incidence patterns and outcomes for hodgkin lymphoma patients in the United States. Adv Hematol..

[21] Shiels MS, Engels EA, Linet MS (2013). The epidemic of non-Hodgkin lymphoma in the United States: disentangling the effect of HIV, 1992-2009. Cancer Epidemiol Biomarkers Prev..

[22] Chihara D, Ito H, Matsuda T, Clarke CA, Li J, Hall HI (2014). Differences in incidence and trends of haematological malignancies in Japan and the United States. Br J Haematol..

[23] Lee H, Park HJ, Park EH, Ju HY, Oh CM, Kong HJ (2018). Nationwide statistical analysis of lymphoid malignancies in Korea. Cancer Res Treat..

[24] Swerdlow SH, Campo E, Pileri SA, Harris NL, Stein H, Siebert R (2016). The 2016 revision of the World Health Organization classification of lymphoid neoplasms. Blood..

[25] Chang ET, Canchola AJ, Cockburn M, Lu Y, Wang SS, Bernstein L (2011). Adulthood residential ultraviolet radiation, sun sensitivity, dietary vitamin D, and risk of lymphoid malignancies in the California Teachers Study. Blood..

[26] Wang SS, Flowers CR, Kadin ME, Chang ET, Hughes AM, Ansell SM (2014). Medical history, lifestyle, family history, and occupational risk factors for peripheral T-cell lymphomas: the InterLymph Non-Hodgkin Lymphoma Subtypes Project. J Natl Cancer Inst Monogr..

[27] Xiong G, Zhang B, Huang MY, Zhou H, Chen LZ, Feng QS (2014). Epstein-Barr virus (EBV) infection in Chinese children: a retrospective study of age-specific prevalence. PLoS One..

[28] Liang X, Bi S, Yang W, Wang L, Cui G, Cui F (2009). Epidemiological serosurvey of hepatitis B in China--declining HBV prevalence due to hepatitis B vaccination. Vaccine..

[29] Bassig BA, Willhauck-Fleckenstein M, Shu XO, Koh WP, Gao YT, Purdue MP (2018). Serologic markers of viral infection and risk of non-Hodgkin lymphoma: a pooled study of three prospective cohorts in China and Singapore. Int J Cancer..

[30] Chen W, Xia C, Zheng R, Zhou M, Lin C, Zeng H (2019). Disparities by province, age, and sex in site-specific cancer burden attributable to 23 potentially modifiable risk factors in China: a comparative risk assessment. Lancet Glob Health..

