# Supplementary material for: Burden of lymphoma in China, 2006–2016: an analysis of the Global Burden of Disease Study 2016
Source: J Hematol Oncol. 2019 Nov 19;12:115. doi: 10.1186/s13045-019-0785-7 (PMC6862726; doi:10.1186/s13045-019-0785-7)
Supplement: Supplementary file 1 — Table S1. Age-standardized incidence rates of lymphoma by age and sex in 2016 (per 100,000). Table S2. Age-standardized mortality rates of lymphoma by age and sex in 2016 (per 100,000). Table S3. Age-standardized incidence, mortality and prevalence rates of lymphoma by province of China in 2016 (per 100,000). Figure S1. Relation between lymphoma burden and socio-demographic index (SDI) at provincial level (A) age-standardized incidence rate (ASIR) of Hodgkin lymphoma (HL) vs. SDI, (B) age-standardized mortality rate (ASMR) of HL vs. SDI, (C) age-standardized prevalence rate (ASPR) of HL vs. SDI, (D) age-standardized disability-adjusted life years (DALYs) of HL vs. SDI, (E) ASIR of non-Hodgkin lymphoma (NHL) vs. SDI, (F) ASMR of NHL vs. SDI, (G) ASPR of NHL vs. SDI, (H) age-standardized DALYs of NHL vs. SDI. (PDF 687 kb) [file 13045_2019_785_MOESM1_ESM.pdf]

Table S1 Age-standardized incidence rates of lymphoma by age and sex in 2016 (per 100,000 population)

Age groups (year)	Hodgkin lymphoma			Non-Hodgkin lymphoma		
	Both	Male	Female	Both	Male	Female
0	0.28 (0.16−0.40)	0.31 (0.18−0.48)	0.25 (0.10−0.39)	0.00	0.00	0.00
1−	0.22 (0.13−0.31)	0.24 (0.15−0.38)	0.19 (0.10−0.28)	1.26 (1.05−1.42)	1.34 (1.06−1.60)	1.17 (0.93−1.39)
5−	0.19 (0.13−0.27)	0.25 (0.15−0.41)	0.12 (0.07−0.16)	0.82 (0.68−0.93)	0.96 (0.75−1.12)	0.66 (0.54−0.77)
10−	0.15 (0.11−0.23)	0.20 (0.12−0.35)	0.10 (0.07−0.13)	0.80 (0.68−0.89)	0.90 (0.76−1.06)	0.67 (0.54−0.78)
15−	0.22 (0.16−0.32)	0.27 (0.16−0.45)	0.17 (0.12−0.26)	0.87 (0.77−1.00)	1.04 (0.92−1.24)	0.68 (0.56−0.83)
20−	0.36 (0.27−0.53)	0.44 (0.27−0.74)	0.27 (0.20−0.41)	0.98 (0.84−1.07)	1.18 (0.98−1.32)	0.75 (0.63−0.90)
25−	0.32 (0.24−0.45)	0.38 (0.24−0.63)	0.26 (0.18−0.38)	1.23 (1.03−1.34)	1.42 (1.13−1.60)	1.02 (0.87−1.16)
30−	0.33 (0.24−0.47)	0.42 (0.26−0.67)	0.23 (0.16−0.33)	1.80 (1.49−1.97)	2.06 (1.61−2.31)	1.53 (1.28−1.76)
35−	0.26 (0.20−0.38)	0.32 (022−0.52)	0.19 (0.14−0.30)	2.22 (1.86−2.45)	2.58 (2.04−2.93)	1.85 (1.48−2.16)

40−	0.29<br>(0.21−0.39)	0.37<br>(0.25−0.58)	0.19<br>(0.14−0.28)	3.29<br>(2.76−3.62)	4.06<br>(3.22−4.58)	2.49<br>(2.06−2.89)
45−	0.45<br>(0.33−0.62)	0.65<br>(0.44−1.00)	0.24<br>(0.16−0.33)	5.62<br>(4.45−6.20)	7.35<br>(5.27−8.38)	3.84<br>(3.35−4.34)
50−	0.69<br>(0.50−0.93)	0.98<br>(0.65−1.47)	0.38<br>(0.23−0.53)	8.21<br>(6.52−8.98)	10.63<br>(7.67−11.86)	5.72<br>(4.83−6.41)
55−	0.85<br>(0.61−1.14)	1.32<br>(0.87−1.88)	0.37<br>(0.24−0.53)	10.37<br>(8.05−11.32)	13.72<br>(9.77−15.34)	6.91<br>(5.77−7.64)
60−	1.20<br>(0.85−1.64)	1.92<br>(1.25−2.75)	0.47<br>(0.30−0.68)	12.61<br>(10.10−13.71)	15.85<br>(11.42−17.66)	9.32<br>(8.04−10.30)
65−	1.28<br>(0.95−1.68)	2.00<br>(1.36−2.79)	0.57<br>(0.40−0.90)	14.62<br>(11.45−15.93)	19.09<br>(13.58−21.32)	10.22<br>(8.62−11.26)
70−	1.33<br>(0.97−1.79)	2.07<br>(1.41−2.95)	0.60<br>(0.44−0.91)	16.78<br>(13.49−18.14)	21.48<br>(15.91−23.64)	12.18<br>(10.24−13.58)
75−	1.25<br>(0.90−1.56)	1.80<br>(1.20−2.36)	0.74<br>(0.51−1.08)	16.00<br>(13.50−17.22)	20.03<br>(15.55−22.03)	12.27<br>(10.53−13.51)
80−	1.12<br>(0.80−1.43)	1.53<br>(0.97−2.04)	0.78<br>(0.49 −1.14)	14.11<br>(11.89−15.31)	17.65<br>(13.56−19.61)	11.08<br>(9.52−12.12)
85−	1.14<br>(0.84−1.50)	1.69<br>(1.15−2.27)	0.77<br>(0.50−1.17)	15.18<br>(13.25−16.43)	19.39<br>(15.04−21.94)	12.36<br>(10.81−13.43)
90−	1.05<br>(0.68−1.29)	1.63<br>(0.98−2.18)	0.78<br>(0.43−1.05)	17.14<br>(15.73−18.88)	21.22<br>(17.39−25.16)	15.12<br>(13.39−16.68)

95−	1.20<br>(0.75−1.46)	1.95<br>(1.16−2.57)	0.76<br>(0.50−1.22)	21.02<br>(19.12−23.14)	28.13<br>(22.29−33.86)	18.55<br>(15.86−20.33)

Data in parentheses are 95% uncertainty intervals.

Table S2 Age-standardized mortality rates of lymphoma by age and sex in 2016 (per 100,000 population)

Age groups (year)	Hodgkin lymphoma			Non-Hodgkin lymphoma		
	Both	Male	Female	Both	Male	Female
0	0.05 (0.04−0.07)	0.07 (0.05−0.09)	0.04 (0.02−0.05)	0.70 (0.57−0.83)	0.73 (0.55−0.88)	0.67 (0.53−0.92)
1−	0.03 (0.02−0.04)	0.04 (0.03−0.06)	0.02 (0.01−0.03)	0.42 (0.35−0.52)	0.47 (0.38−0.59)	0.36 (0.29−0.48)
5−	0.03 (0.03−0.05)	0.05 (0.04−0.07)	0.02 (0.01−0.02)	0.32 (0.27−0.37)	0.39 (0.32−0.47)	0.24 (0.20−0.29)
10−	0.03 (0.03−0.05)	0.05 (0.04−0.07)	0.02 (0.01−0.03)	0.36 (0.32−0.41)	0.43 (0.37−0.51)	0.28 (0.24−0.34)
15−	0.05 (0.04−0.07)	0.06 (0.05−0.10)	0.03 (0.02−0.05)	0.41 (0.36−0.47)	0.51 (0.45−0.61)	0.29 (0.24−0.36)
20−	0.06 (0.05−0.09)	0.08 (0.06−0.12)	0.04 (0.03−0.06)	0.54 (0.46−0.61)	0.67 (0.55−0.76)	0.39 (0.32−0.49)
25−	0.07 (0.06−0.10)	0.09 (0.07−0.13)	0.05 (0.04−0.08)	0.61 (0.51−0.67)	0.72 (0.57−0.82)	0.48 (0.42−0.56)
30−	0.09 (0.07−0.12)	0.12 (0.08−0.17)	0.05 (0.04−0.08)	0.70 (0.58−0.77)	0.85 (0.66−0.94)	0.54 (0.47−0.64)
35−	0.08 (0.07−0.11)	0.11 (0.08−0.16)	0.06 (0.04−0.09)	0.85 (0.72−0.94)	1.05 (0.84−1.17)	0.65 (0.54−0.77)

40−	0.11<br>(0.09−0.15)	0.14<br>(0.11−0.22)	0.07<br>(0.05−0.11)	1.30<br>(1.09−1.44)	1.70<br>(1.34−1.93)	0.89<br>(0.75−1.03)
45−	0.16<br>(0.14−0.23)	0.23<br>(0.18−0.35)	0.09<br>(0.06−0.13)	2.19<br>(1.70−2.41)	3.05<br>(2.15−3.46)	1.30<br>(1.15−1.46)
50−	0.24<br>(0.19−0.33)	0.32<br>(0.24−0.46)	0.16<br>(0.10−0.24)	3.41<br>(2.63−3.76)	4.78<br>(3.37−5.36)	2.00<br>(1.73−2.25)
55−	0.35<br>(0.27−0.46)	0.50<br>(0.37−0.69)	0.20<br>(0.14−0.30)	5.04<br>(3.84−5.57)	7.14<br>(4.98−8.03)	2.87<br>(2.43−3.20)
60−	0.53<br>(0.42−0.70)	0.75<br>(0.53−1.05)	0.29<br>(0.19−0.44)	6.91<br>(5.47−7.57)	9.25<br>(6.76−10.33)	4.53<br>(3.95−5.04)
65−	0.72<br>(0.58−0.97)	1.02<br>(0.74−1.42)	0.43<br>(0.31−0.69)	9.59<br>(7.54−10.50)	13.06<br>(9.24−14.57)	6.17<br>(5.28−6.86)
70−	0.91<br>(0.70−1.23)	1.31<br>(0.94−1.94)	0.52<br>(0.38−0.80)	12.10<br>(9.73−13.14)	16.17<br>(12.03−17.82)	8.12<br>(6.95−9.15)
75−	0.94<br>(0.70−1.19)	1.23<br>(0.83−1.63)	0.66<br>(0.47−0.99)	13.01<br>(10.94−14.11)	17.18<br>(13.65−19.02)	9.17<br>(7.94−10.20)
80−	1.10<br>(0.81−1.44)	1.41<br>(0.91−1.90)	0.84<br>(0.53−1.23)	14.09<br>(11.83−15.48)	18.66<br>(14.56−21.10)	10.16<br>(8.78−11.29)
85−	1.09<br>(0.83−1.45)	1.50<br>(1.04−2.06)	0.83<br>(0.54−1.25)	14.61<br>(12.61−16.08)	19.97<br>(15.54−22.79)	11.02<br>(9.66−12.13)
90−	0.98<br>(0.68−1.23)	1.37<br>(0.89−1.87)	0.78<br>(0.45−1.07)	15.61<br>(14.20−17.52)	21.04<br>(17.58−25.32)	12.92<br>(11.47−14.23)

95−	1.14<br>(0.72−1.37)	1.62<br>(1.01−2.05)	0.97<br>(0.53−1.27)	18.85<br>(17.21−21.19)	27.89<br>(22.80−34.36)	15.72<br>(13.77−17.44)

Data in parentheses are 95% uncertainty intervals.

Table S3 Age-standardized incidence, mortality and prevalence rates of lymphoma by province of China in 2016 (per 100,000 population)

Province	Hodgkin lymphoma			Non-Hodgkin lymphoma		
	Incidence	Mortality	Prevalence	Incidence	Mortality	Prevalence
Anhui	0.34 (0.24−0.45)	0.22 (0.16−0.28)	1.07 (0.74−1.51)	5.10 (3.44−5.89)	3.00 (2.08−3.57)	17.38 (11.37−20.38)
Beijing	0.77 (0.53−1.01)	0.11 (0.07−0.13)	3.50 (2.39−4.68)	7.55 (6.46−8.30)	2.49 (1.93−2.93)	30.31 (25.90−33.50)
Chongqing	0.32 (0.23−0.56)	0.16 (0.12−0.26)	1.16 (0.77−2.11)	3.07 (2.81−3.33)	2.17 (1.87−2.51)	9.86 (8.98−10.72)
Fujian	0.46 (0.29−0.67)	0.18 (0.13−0.22)	1.75 (1.05−2.71)	3.91 (3.24−4.35)	2.42 (2.00−2.81)	13.26 (10.76−14.83)
Gansu	0.24 (0.18−0.43)	0.18 (0.13−0.32)	0.73 (0.52−1.30)	2.51 (2.18−2.73)	2.17 (1.87−2.51)	7.21 (6.12−7.91)
Guangdong	0.55 (0.39−0.83)	0.14 (0.12−0.19)	2.30 (1.56−3.57)	5.72 (5.17−6.80)	2.06 (1.76−2.53)	22.43 (20.19−26.53)
Guangxi	0.45 (0.34−0.66)	0.24 (0.19−0.33)	1.58 (1.12−2.50)	3.48 (2.77−3.87)	2.51 (2.04−2.92)	10.98 (8.65−12.29)
Guizhou	0.24 (0.15−0.52)	0.20 (0.12−0.45)	0.70 (0.43−1.46)	2.81 (2.19−3.12)	2.58 (2.11−3.03)	7.86 (5.89−8.76)
Hainan	0.33 (0.21−0.64)	0.15 (0.10−0.29)	1.24 (0.73−2.44)	3.04 (2.79−3.48)	2.06 (1.78−2.52)	9.99 (9.11−11.18)

Hebei	0.56<br>(0.34−0.81)	0.24<br>(0.15−0.29)	2.12<br>(1.24−3.26)	3.78<br>(3.05−4.20)	2.40<br>(1.86−2.80)	12.58<br>(10.04−14.04)
Heilongjiang	0.40<br>(0.29−0.64)	0.19<br>(0.15−0.27)	1.47<br>(1.02−2.40)	3.25<br>(2.95−3.71)	2.26<br>(1.95−2.60)	10.51<br>(9.42−11.75)
Henan	0.46<br>(0.32−0.68)	0.22<br>(0.17−0.28)	1.67<br>(1.10−2.65)	3.26<br>(2.86−3.65)	2.22<br>(1.89−2.57)	10.48<br>(9.17−11.59)
Hubei	0.44<br>(0.30−0.62)	0.21<br>(0.16−0.26)	1.58<br>(1.03−2.42)	5.14<br>(4.24−5.63)	2.56<br>(2.12−2.92)	18.57<br>(15.15−20.53)
Hunan	0.33<br>(0.23−0.56)	0.17<br>(0.13−0.28)	1.18<br>(0.78−2.10)	4.16<br>(2.93−4.73)	2.96<br>(2.10−3.46)	13.25<br>(9.04−15.17)
Inner Mongolia	0.36<br>(0.24−0.68)	0.14<br>(0.11−0.24)	1.38<br>(0.86−2.73)	3.91<br>(3.24−4.33)	2.46<br>(1.98−2.89)	13.20<br>(10.83−14.65)
Jiangsu	0.61<br>(0.29−0.95)	0.19<br>(0.09−0.25)	2.51<br>(1.14−4.13)	4.91<br>(4.00−5.42)	2.52<br>(1.97−2.94)	17.72<br>(14.34−19.68)
Jiangxi	0.34<br>(0.26−0.50)	0.22<br>(0.17−0.28)	1.14<br>(0.81−1.75)	3.16<br>(2.47−3.50)	2.53<br>(2.01−2.92)	9.63<br>(7.35−10.75)
Jilin	0.40<br>(0.29−0.60)	0.18<br>(0.15−0.25)	1.49<br>(1.01−2.38)	3.60<br>(2.92−3.96)	2.40<br>(1.89−2.79)	11.83<br>(9.61−13.07)
Liaoning	0.57<br>(0.32−0.87)	0.20<br>(0.12−0.25)	2.26<br>(1.21−3.66)	6.27<br>(4.94−7.02)	2.70<br>(2.05−3.16)	23.72<br>(18.73−26.72)
Ningxia	0.27<br>(0.19−0.47)	0.17<br>(0.12−0.29)	0.90<br>(0.61−1.59)	2.81<br>(2.58−3.04)	2.23<br>(1.93−2.57)	8.56<br>(7.67−9.25)

Qinghai	0.31<br>(0.19−0.69)	0.22<br>(0.13−0.48)	0.99<br>(0.57−2.19)	3.05<br>(2.54−3.33)	2.60<br>(2.18−3.01)	8.90<br>(7.25−9.73)
Shaanxi	0.32<br>(0.22−0.56)	0.16<br>(0.12−0.28)	1.14<br>(0.74−2.11)	2.91<br>(2.66−3.22)	2.10<br>(1.80−2.45)	9.26<br>(8.41−10.12)
Shandong	0.56<br>(0.34−0.83)	0.19<br>(0.13−0.23)	2.21<br>(1.30−3.41)	4.18<br>(3.57−4.60)	2.27<br>(1.86−2.63)	14.73<br>(12.60−16.34)
Shanghai	0.66<br>(0.45−0.93)	0.10<br>(0.07−0.12)	2.95<br>(1.99−4.27)	8.36<br>(6.04−9.40)	2.84<br>(1.98−3.38)	33.47<br>(24.10−37.84)
Shanxi	0.33<br>(0.23−0.57)	0.15<br>(0.12−0.26)	1.22<br>(0.80−2.16)	3.56<br>(3.10−3.87)	2.41<br>(2.01−2.79)	11.57<br>(9.94−12.66)
Sichuan	0.37<br>(0.29−0.54)	0.22<br>(0.18−0.31)	1.25<br>(0.92−1.96)	3.33<br>(2.73−3.65)	2.55<br>(2.10−2.98)	10.20<br>(8.21−11.27)
Tianjin	0.69<br>(0.47−1.02)	0.13<br>(0.10−0.17)	3.02<br>(2.03−4.55)	7.57<br>(6.45−8.39)	2.74<br>(2.20−3.19)	29.92<br>(25.51−33.30)
Tibet	0.36<br>(0.24−0.74)	0.33<br>(0.21−0.69)	1.00<br>(0.68−2.03)	3.48<br>(2.32−4.00)	3.49<br>(2.34−4.22)	9.65<br>(6.18−11.20)
Xinjiang	0.52<br>(0.35−0.95)	0.24<br>(0.18−0.39)	1.94<br>(1.22−3.70)	3.25<br>(2.94−4.13)	2.25<br>(1.92−2.84)	10.62<br>(9.55−13.27)
Yunnan	0.31<br>(0.23−0.55)	0.22<br>(0.16−0.40)	0.96<br>(0.69−1.71)	2.71<br>(2.31−2.96)	2.31<br>(1.98−2.68)	7.87<br>(6.62−8.62)
Zhejiang	0.60<br>(0.27−0.97)	0.19<br>(0.10−0.25)	2.44<br>(1.06−4.18)	5.27<br>(4.61−5.79)	2.14<br>(1.80−2.50)	20.18<br>(17.56−22.28)

Hong Kong*	0.45<br>(0.32−0.62)	0.09<br>(0.07−0.12)	1.96<br>(1.36−2.77)	7.78<br>(4.85−8.98)	3.43<br>(2.20−4.08)	29.47<br>(18.67−34.03)
Macao*	0.32<br>(0.21−0.63)	0.06<br>(0.04−0.11)	1.43<br>(0.91−2.81)	5.22<br>(3.44−6.45)	2.21<br>(1.42−2.77)	19.68<br>(13.08−24.60)

*special administrative regions.

Data in parentheses are 95% uncertainty intervals.

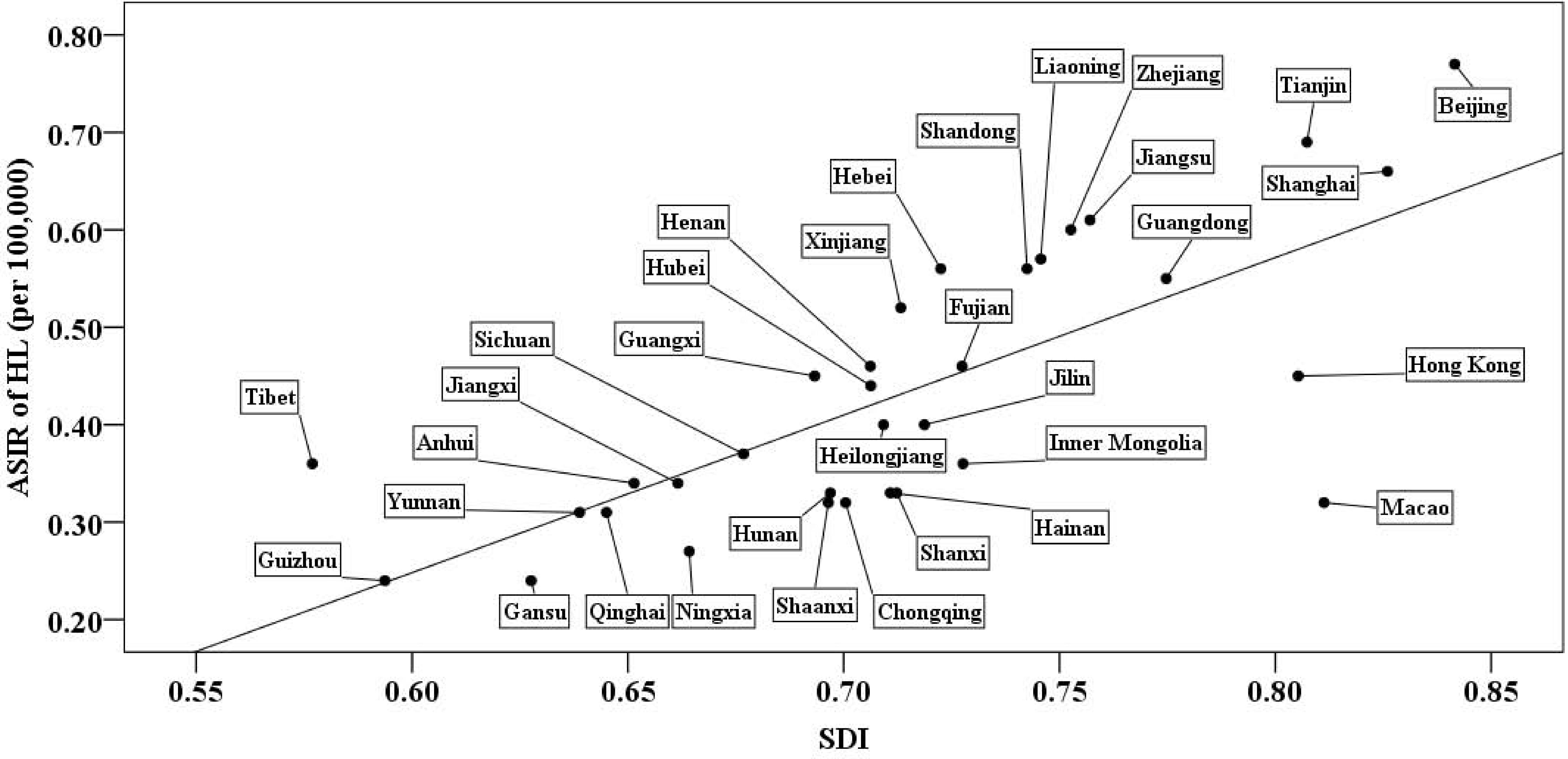
ASIR of HL (per 100,000)
0.80
0.70
0.60
0.50
0.40
0.30
0.20
SDI
0.55
0.60
0.65
0.70
0.75
0.80
0.85
Liaoning
Zhejiang
Tianjin
Beijing
Shandong
Jiangsu
Shanghai
Hebei
Henan
Xinjiang
Guangdong
Hubei
Fujian
Sichuan
Guangxi
Hong Kong
Tibet
Jiangxi
Jilin
Anhui
Inner Mongolia
Heilongjiang
Yunnan
Macao
Hainan
Hunan
Shanxi
Guizhou
Gansu
Qinghai
Ningxia
Shaanxi
Chongqing

(A)

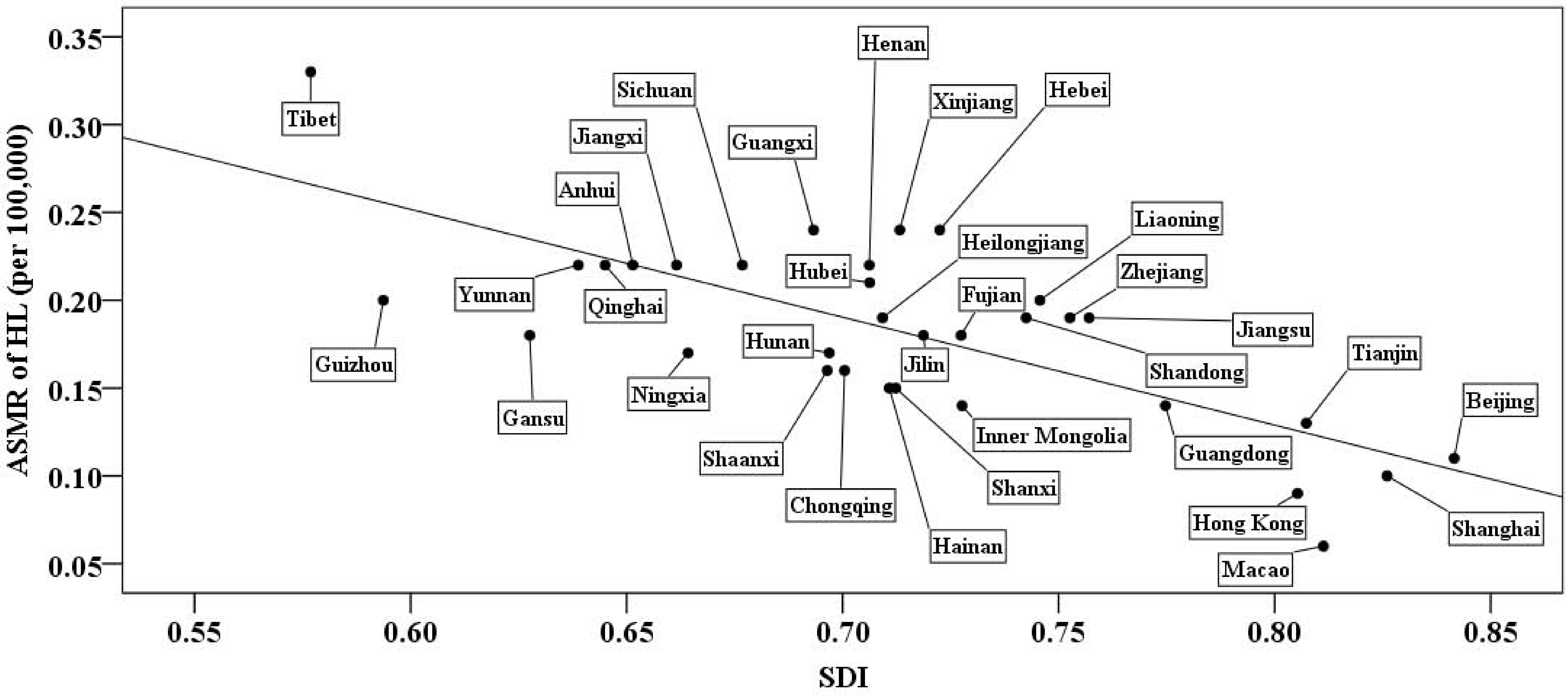
ASMR of HL (per 100,000)
0.35
0.30
0.25
0.20
0.15
0.10
0.05
0.55
0.60
0.65
0.70
0.75
0.80
0.85
SDI
Tibet
Guizhou
Gansu
Yunnan
Qinghai
Anhui
Jiangxi
Sichuan
Ningxia
Guangxi
Hunan
Shaanxi
Chongqing
Henan
Hubei
Xinjiang
Hebei
Heilongjiang
Jilin
Hainan
Shanxi
Fujian
Inner Mongolia
Liaoning
Zhejiang
Jiangsu
Shandong
Guangdong
Tianjin
Beijing
Hong Kong
Macao
Shanghai

(B)

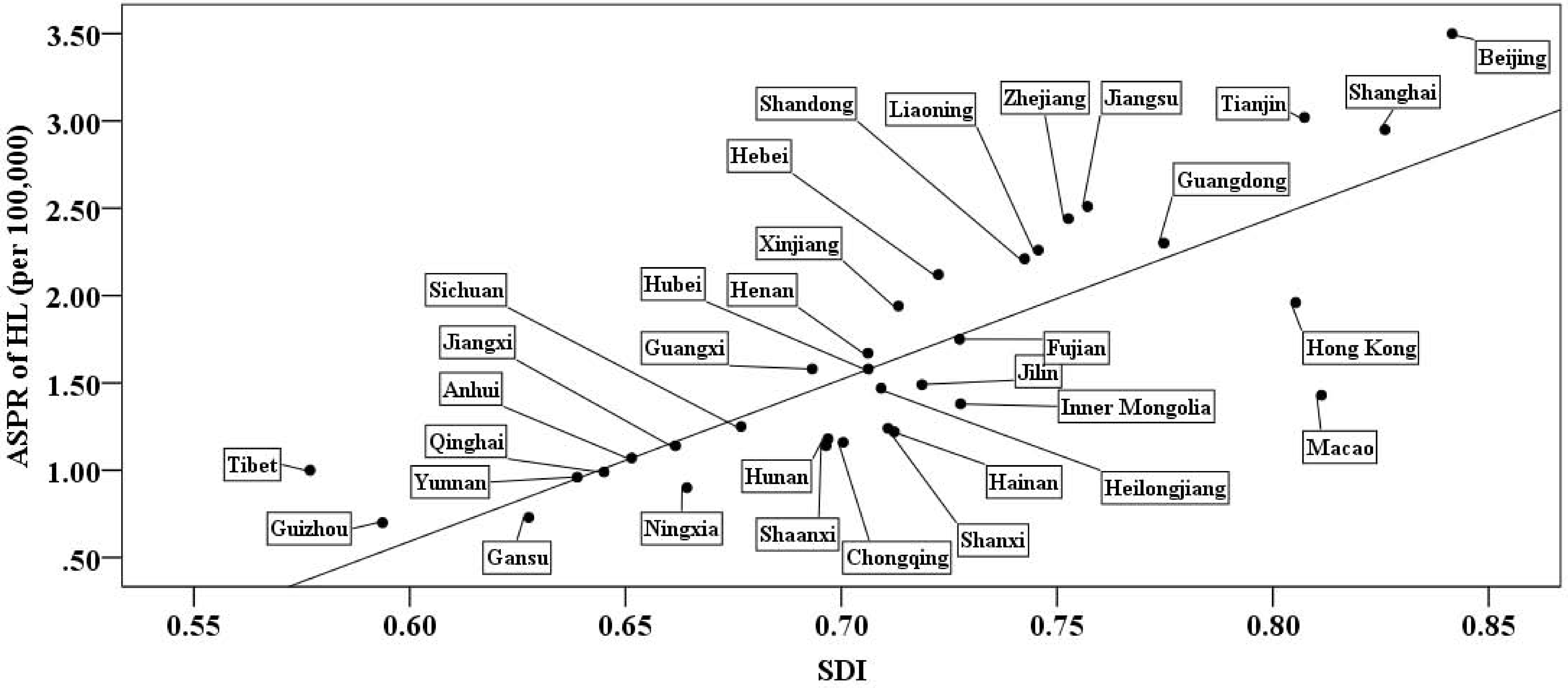
ASPR of HL (per 100,000)
SDI
3.50
3.00
2.50
2.00
1.50
1.00
.50
0.55
0.60
0.65
0.70
0.75
0.80
0.85
Beijing
Shanghai
Tianjin
Jiangsu
Zhejiang
Liaoning
Shandong
Hebei
Guangdong
Xinjiang
Hubei
Henan
Sichuan
Jiangxi
Guangxi
Fujian
Jilin
Inner Mongolia
Hong Kong
Macao
Anhui
Qinghai
Yunnan
Tibet
Guizhou
Gansu
Ningxia
Hunan
Shaanxi
Chongqing
Shanxi
Hainan
Heilongjiang

(C)

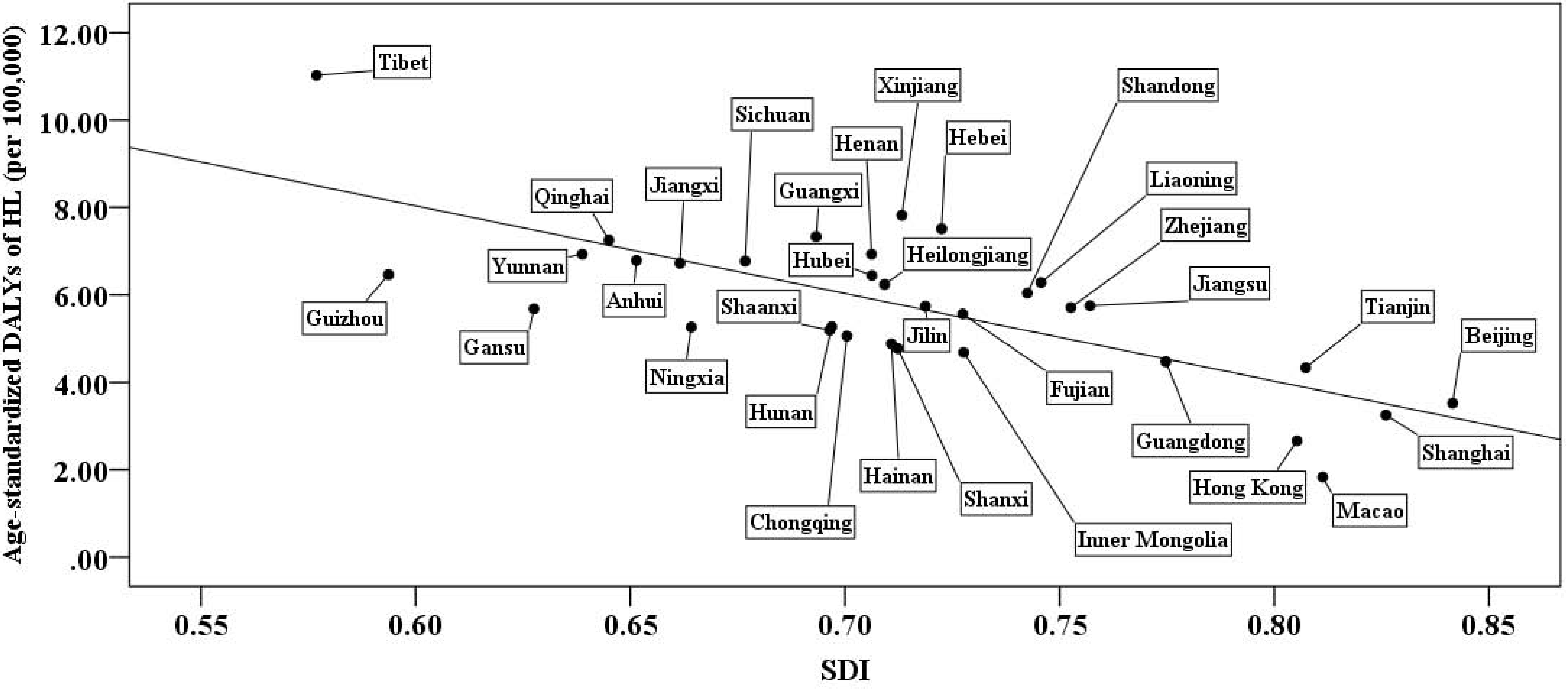

Age-standardized DALYs of HL (per 100,000)
12.00
10.00
8.00
6.00
4.00
2.00
.00
0.55
0.60
0.65
0.70
0.75
0.80
0.85
SDI
Tibet
Sichuan
Xinjiang
Shandong
Henan
Hebei
Jiangxi
Guangxi
Liaoning
Qinghai
Zhejiang
Hubei
Heilongjiang
Yunnan
Jiangsu
Anhui
Guizhou
Shaanxi
Tianjin
Jilin
Beijing
Gansu
Ningxia
Fujian
Hunan
Guangdong
Shanghai
Hainan
Hong Kong
Shanxi
Macao
Chongqing
Inner Mongolia

(D)

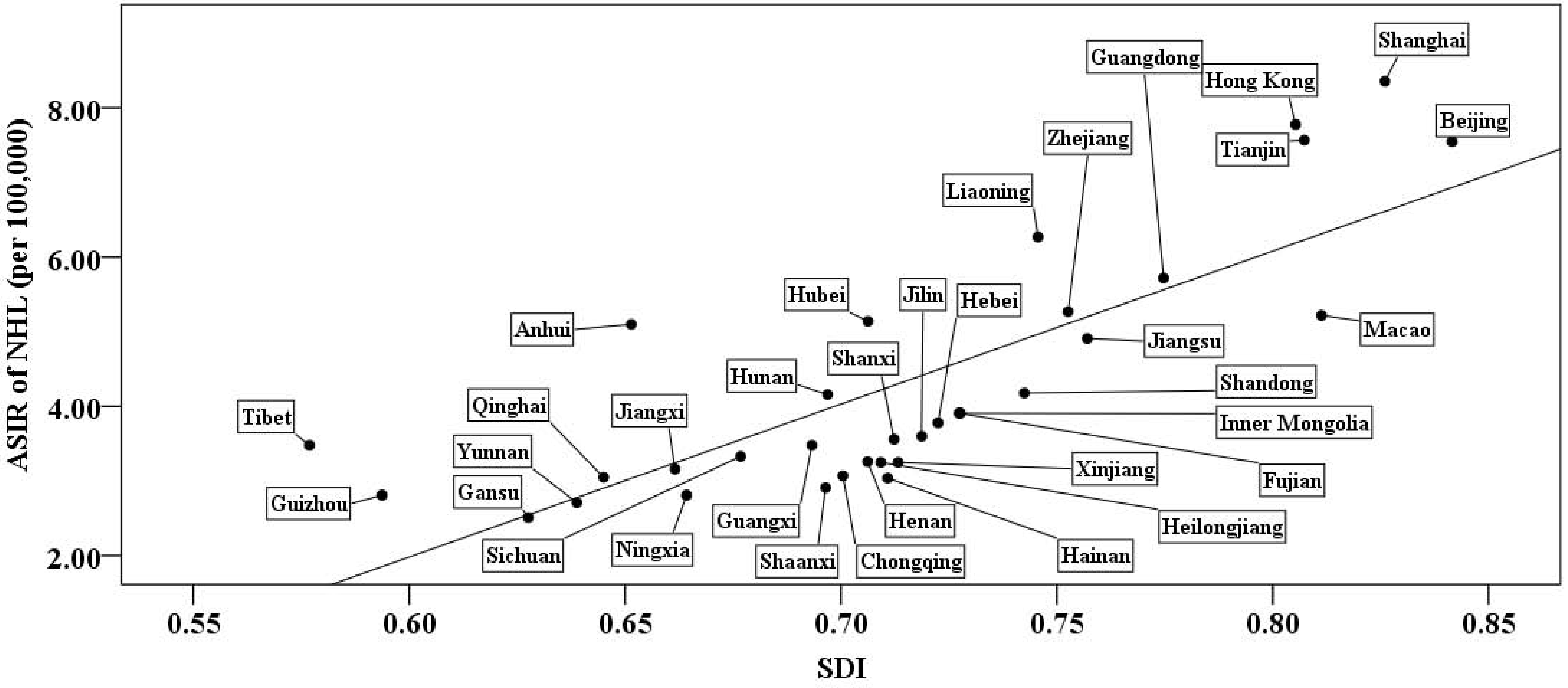
ASIR of NHL (per 100,000)
SDI
8.00
6.00
4.00
2.00
0.55
0.60
0.65
0.70
0.75
0.80
0.85
Shanghai
Guangdong
Hong Kong
Beijing
Tianjin
Zhejiang
Liaoning
Hubei
Jilin
Hebei
Anhui
Macao
Jiangsu
Shanxi
Hunan
Shandong
Qinghai
Jiangxi
Inner Mongolia
Tibet
Yunnan
Xinjiang
Fujian
Gansu
Guizhou
Guangxi
Henan
Heilongjiang
Sichuan
Ningxia
Shaanxi
Chongqing
Hainan

(E)

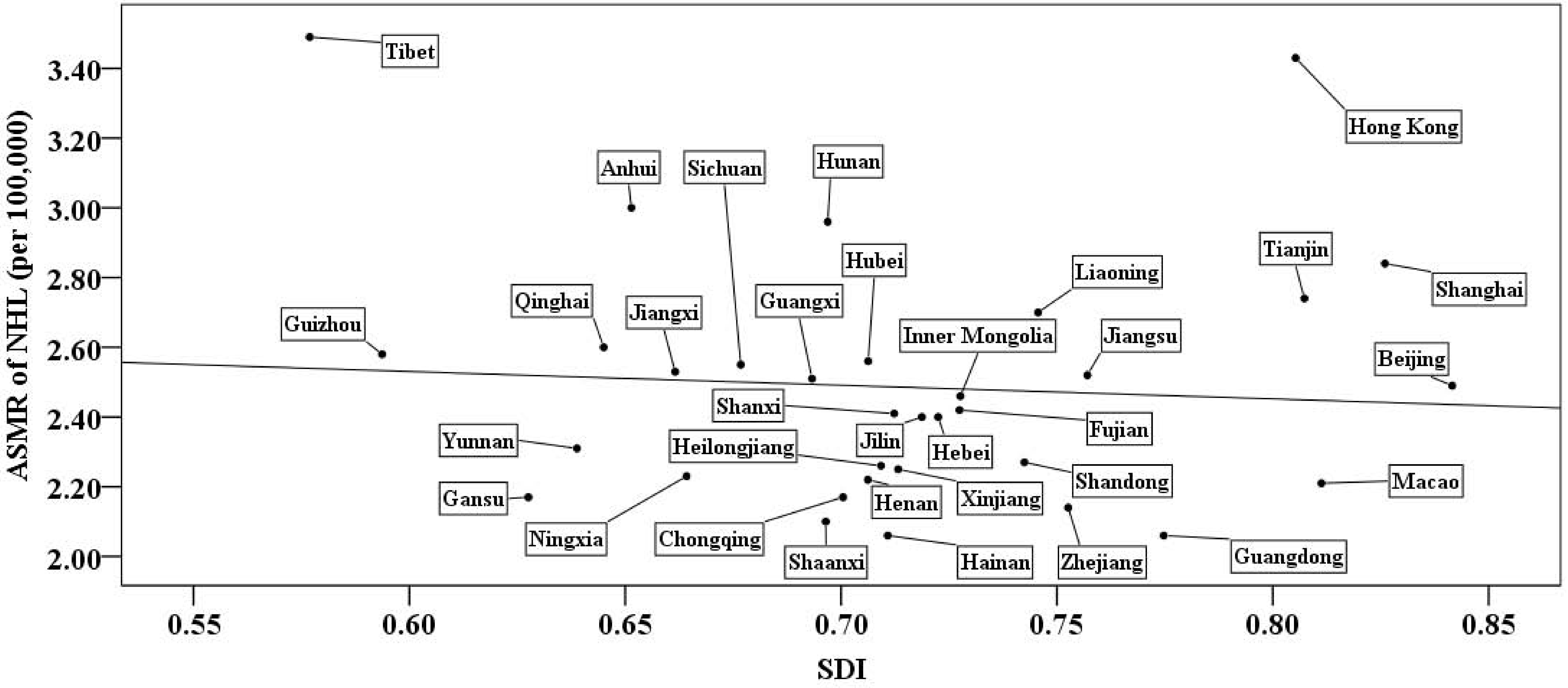

ASMR of NHL (per 100,000)
3.40
3.20
3.00
2.80
2.60
2.40
2.20
2.00
0.55
0.60
0.65
0.70
0.75
0.80
0.85
SDI
Tibet
Hong Kong
Anhui
Sichuan
Hunan
Hubei
Tianjin
Liaoning
Shanghai
Qinghai
Jiangxi
Guangxi
Guizhou
Inner Mongolia
Jiangsu
Beijing
Shanxi
Jilin
Hebei
Fujian
Yunnan
Heilongjiang
Shandong
Macao
Gansu
Henan
Xinjiang
Ningxia
Chongqing
Shaanxi
Hainan
Zhejiang
Guangdong

(F)

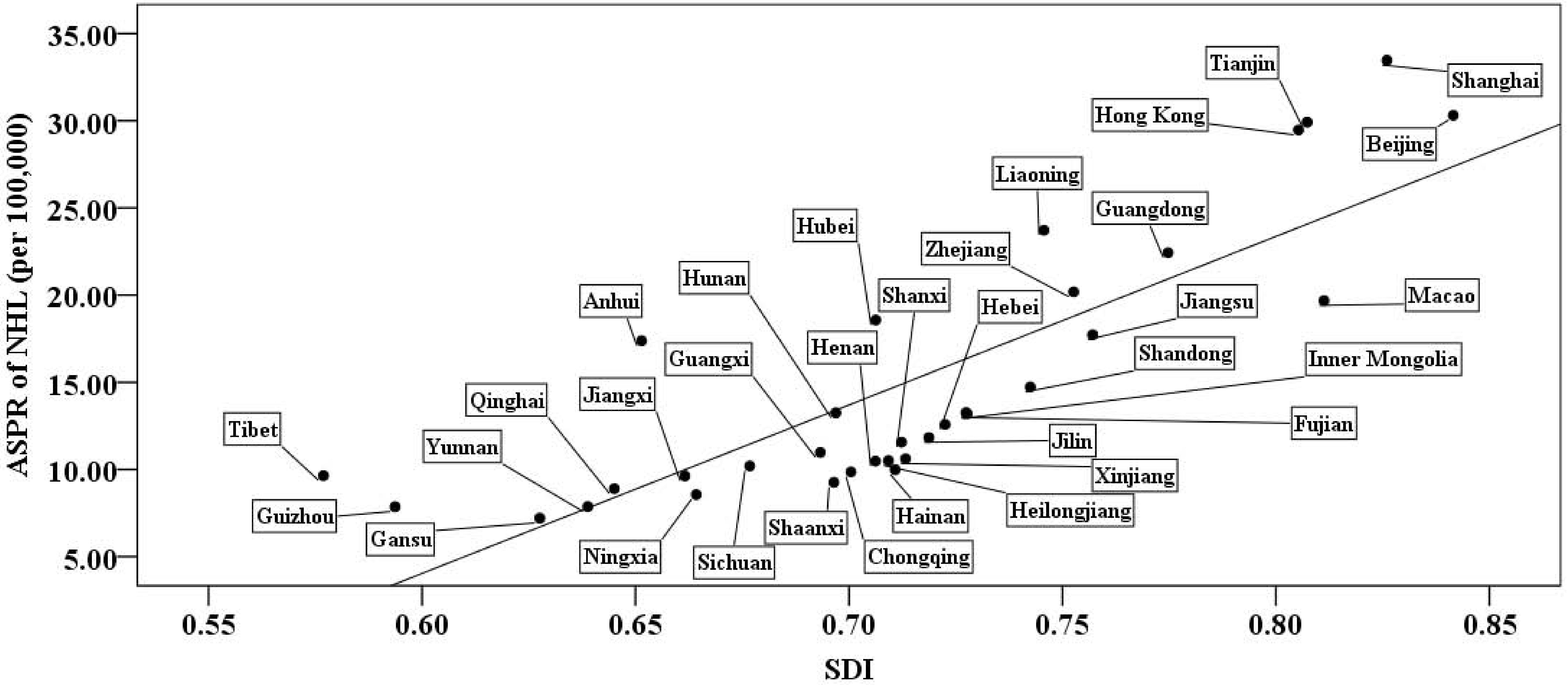
ASPR of NHL (per 100,000)
35.00
30.00
25.00
20.00
15.00
10.00
5.00
SDI
0.55
0.60
0.65
0.70
0.75
0.80
0.85
Tianjin
Shanghai
Hong Kong
Beijing
Liaoning
Guangdong
Hubei
Zhejiang
Hunan
Shanxi
Hebei
Macao
Anhui
Jiangsu
Henan
Guangxi
Shandong
Inner Mongolia
Jiangxi
Qinghai
Fujian
Tibet
Yunnan
Jilin
Xinjiang
Heilongjiang
Guizhou
Hainan
Gansu
Shaanxi
Ningxia
Sichuan
Chongqing

(G)

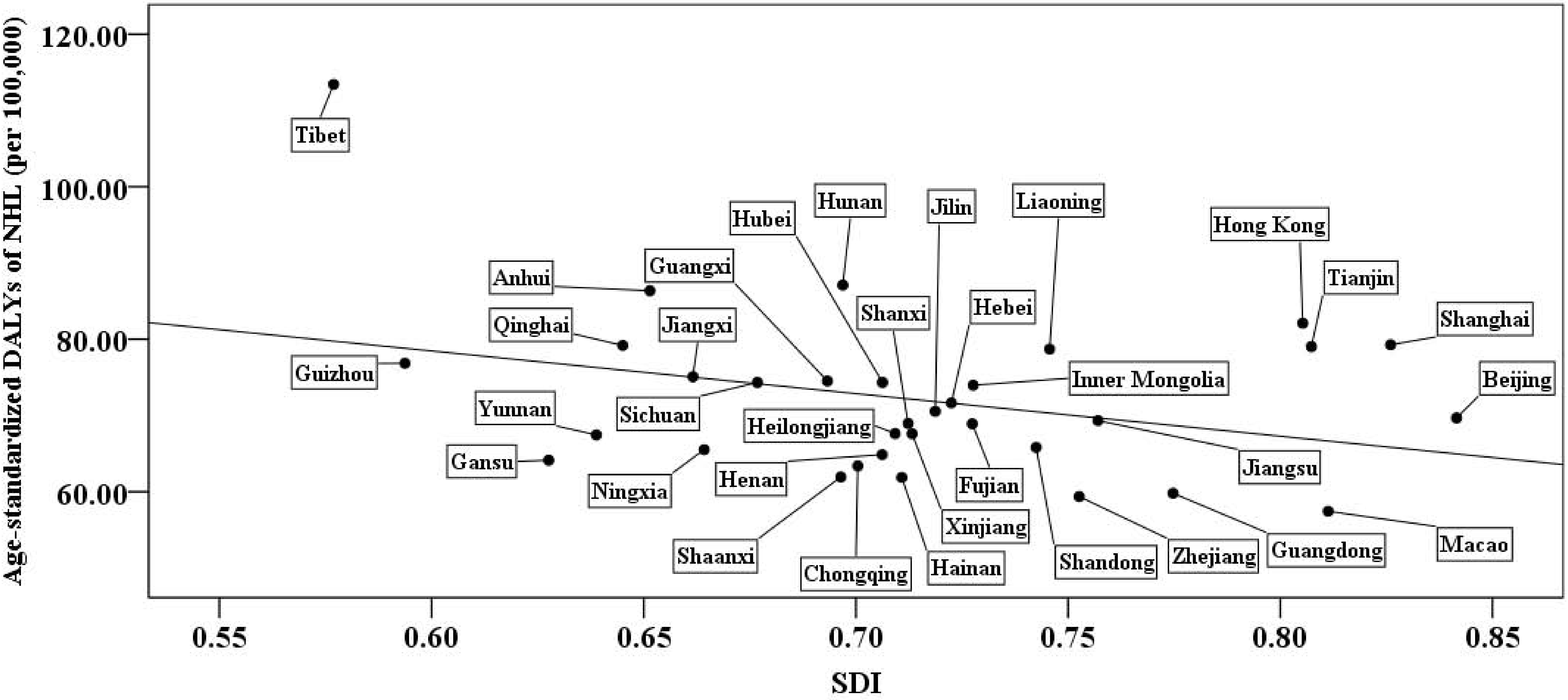

Age-standardized DALYs of NHL (per 100,000)
120.00
100.00
80.00
60.00
Tibet
Hunan
Hubei
Jilin
Liaoning
Hong Kong
Guangxi
Anhui
Tianjin
Shanxi
Hebei
Qinghai
Jiangxi
Shanghai
Guizhou
Inner Mongolia
Beijing
Yunnan
Sichuan
Heilongjiang
Gansu
Jiangsu
Ningxia
Henan
Fujian
Xinjiang
Shaanxi
Chongqing
Hainan
Shandong
Zhejiang
Guangdong
Macao
0.55
0.60
0.65
0.70
0.75
0.80
0.85
SDI

(H)

Figure S1. Relation between lymphoma burden and socio-demographic index (SDI) at provincial level (A) age-standardized incidence rate (ASIR) of Hodgkin lymphoma (HL) vs. SDI, (B) age-standardized mortality rate (ASMR) of HL vs. SDI, (C) age-standardized prevalence rate (ASPR) of HL vs. SDI, (D) age-standardized disability-adjusted life years (DALYs) of HL vs. SDI, (E) ASIR of non-Hodgkin lymphoma (NHL) vs. SDI, (F) ASMR of NHL vs. SDI, (G) ASPR of NHL vs. SDI, (H) age-standardized DALYs of NHL vs. SDI
